# Comprehensive Analysis of N6-Methyladenosine (m6A) Writers, Erasers, and Readers in Cervical Cancer

**DOI:** 10.3390/ijms23137165

**Published:** 2022-06-28

**Authors:** Mateja Condic, Damian J. Ralser, Niklas Klümper, Jörg Ellinger, Maryam Qureischi, Eva K. Egger, Glen Kristiansen, Alexander Mustea, Thore Thiesler

**Affiliations:** 1 Department of Gynecology and Gynecological Oncology, University Hospital Bonn, 53127 Bonn, Germany; damian.ralser@ukbonn.de (D.J.R.); maryam.qureischi@ukbonn.de (M.Q.); eva-katharina.egger@ukbonn.de (E.K.E.); alexander.mustea@ukbonn.de (A.M.); 2 Department of Urology and Pediatric Urology, University Hospital Bonn, 53127 Bonn, Germany; niklas.kluemper@ukbonn.de (N.K.); joerg.ellinger@ukbonn.de (J.E.); 3 Institute of Pathology, University Hospital Bonn, 53127 Bonn, Germany; glen.kristiansen@ukbonn.de (G.K.); thore.thiesler@ukbonn.de (T.T.)

**Keywords:** cervical cancer, m6a, RNA modification, biomarker

## Abstract

There is growing scientific evidence for the crucial role of post-transcriptional RNA modifications in carcinogenesis, progression, metastasis, and drug resistance across various cancer entities. N6-methyladenosine (m6A) is the most abundant type of RNA modification. m6A is coordinated by a dynamic interplay of ‘writers’ (METTL3, METTL4, METTL14, WTAP, KIAA1429), ‘erasers’ (FTO, ALKBH5), and ‘readers’ (HNRNPA2B1, HNRNPC, YTHDC1, YTHDC1, YTHDF1-3). In this study, we comprehensively examined protein and mRNA expression levels of m6A writers, readers, and erasers in two cervical cancer (CC) cohorts (UHB CC cohort, *N* = 118; TCGA CC cohort, *N* = 307) with regard to clinical outcomes. In the UHB CC cohort, high protein expression levels of METTL14 (*p* = 0.016), WTAP (*p* = 0.007), KIAA1439 (*p* < 0.001), ALKBH5 (*p* < 0.001), HNRNPC (*p* = 0.012), YTHDC1 (*p* < 0.001), and YTHDF3 (*p* = 0.004) were significantly associated with a shorter overall survival (OS). In the TCGA CC cohort, mRNA expression levels of *METTL14* (*p* = 0.012), *WTAP* (*p* = 0.041), *KIAA1429* (*p* = 0.016), and *YTHDC1* (*p* = 0.026) showed prognostic values. However, after correction for multiple testing, statistical significance remained only for m6A protein expression levels (*q* < 0.1). Our study points towards dysregulated m6A modification in CC. Hence, m6A might serve as a promising prognostic biomarker and therapeutical target in CC.

## 1. Introduction

Cervical cancer (CC) represents the fourth most common malignancy diagnosed in women worldwide [1]. CC incidence varies substantially depending on the availability of an effective screening program, causing significantly higher incidence and cancer-related deaths in developing countries [2]. The predominant histologic CC subtype is squamous cell carcinoma, accounting for over 80% of all cases. The remaining 20% are mainly attributable to adenocarcinomas and less common histologic subtypes [3,4]. Research has identified infection with human papilloma virus (HPV) as an obligatory cofactor for the development of CC [5]. The use of cervical cytology and HPV co-testing has significantly improved the detection of preinvasive cervical lesions and resulted in the significant decrease in invasive CC incidence [6] HPV vaccination, implemented since the mid-2000s, is expected to lead to further reductions in CC disease rates [7]. For early-stage CC, standard surgical treatment consists of radical hysterectomy. In patients with advanced local disease or presence of histopathologic risk factors, concurrent chemoradiotherapy is an equivalent therapy approach [8]. The prognosis of CC is stage-dependent. While early carcinomas display excellent 5-year survival rates, the prognosis of advanced disease stages is extremely poor. In particular, the treatment of recurrent or metastatic CC is challenging due to a lack of effective therapeutic strategies. In this context, a deeper understanding of CC carcinogenesis, in particular epigenetic regulation mechanisms of oncogenic drivers, might help to discover potential targets for individualized therapy.

Research has implicated post-transcriptional messenger RNA (mRNA) modification to be involved in tumorigenesis, proliferation, angiogenesis, and tumor immunity across different cancer entities [9,10,11]. In this context, N6-methyladenosine (m6A) has been identified as the most common type of mRNA modification. The biological importance of m6A underlines its great potential to be used for diagnostic and therapeutic purposes. The process of m6A is coordinated by three different enzyme groups, designated as ‘writers’ (methylases; METTL 3, METTL 4, METTL 14, WTAP, KIAA1429), ‘erasers’ (demethylases; FTO, ALKBH5), and ‘readers’ (HNRNPA2B1, HNRNPC, YTHDC1, YTHDF1-3). Writers and erasers have opposite functions: while writers transfer S-adenosyl methionine to the RNA base adenine, erasers undo this process. These m6A RNA modifications are recognized by readers to mediate downstream effects [12].

However, little is known about the expression levels of m6A writers, erasers, and readers in CC. In this study, we comprehensively examined protein and mRNA expression levels of m6A writers, readers, and erasers in CC with regard to clinical outcomes.

## 2. Results

Immunohistochemical staining was performed in the UHB CC cohort comprising 118 patients. The mean age of the study cohort was 51.3 (+/− standard deviation (SD) 13.9) years. In total, 83.1% of the patients had squamous histology, and 16.9% were cervical adenocarcinomas. The median follow-up was 77.6 months. Clinicopathologic characteristics of the UHB CC cohort (grading, lymph node involvement, tumor stage according to FIGO, HPV status) are shown in Table 1.

In the UHB CC cohort, expression of all different m6A writers, readers, and erasers was identified (Supplementary Figures S2–S8). The proteins involved in m6A functions were present in different cell compartments, reflecting the diversity of RNA metabolism. Writers were typically observed in the nucleus. Congruently, METTL3, METTL14, WTAP, and KIAA1429 displayed strong nuclear staining. Likewise, immunohistochemical analysis revealed a strong nuclear staining for the eraser FTO and the two readers HNRNPC und HNRNPA2B1. In contrast, the readers YTHDF1, YTHDF2, and YTHDF3, as well as the writer METTL4, were detected in the cytoplasm (Table 2).

**Figure 1:**
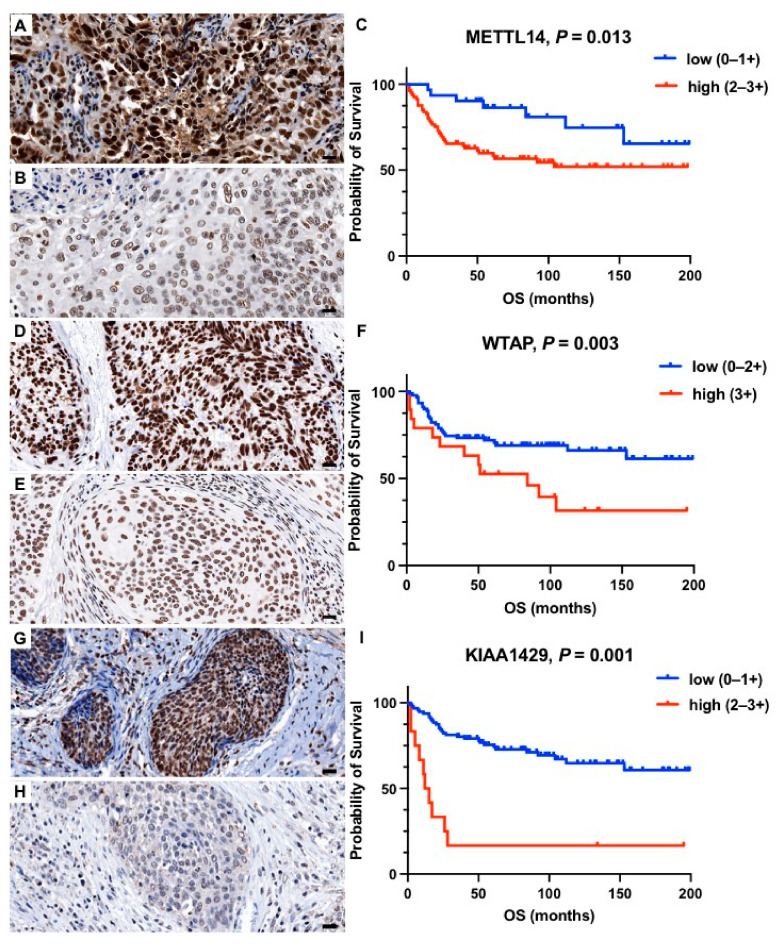
Representative histology sections show high (**A**,**D**,**G**) and low (**B**,**E**,**H**) expression levels of METTL14, WTAP, and KIAA1429 visualized by immunohistochemistry; hematoxylin (blue) was used for nuclear staining (bright field image, 400× magnification). Kaplan–Meier estimates show a significantly shorter overall survival (*p* < 0.05) in patients with high expression of (**C**) METTL14, (**F**) WTAP, and (**I**) KIAA1429. Scale bar = 20 µm.

**Figure 2:**
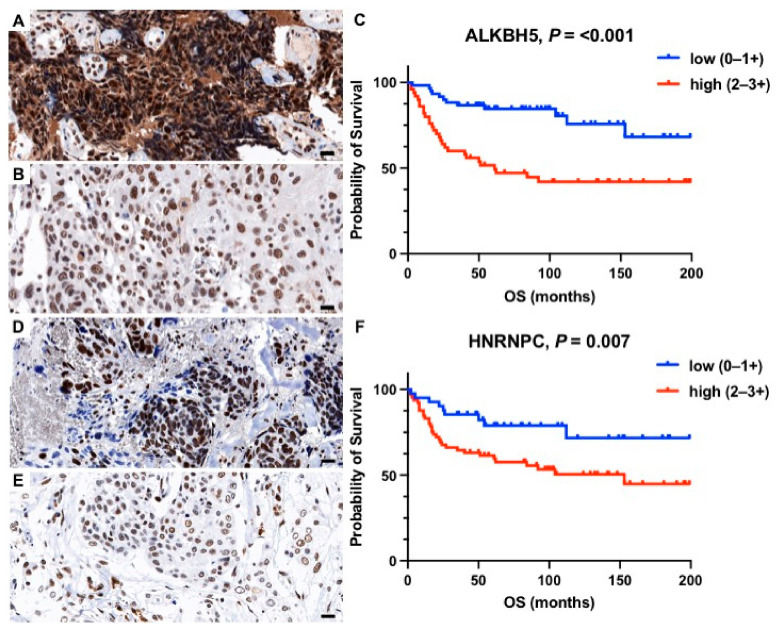
Representative histology sections show high (**A**,**D**) and low (**B**,**E**) expression levels of ALKBH5 and HNRNPC visualized by immunohistochemistry; hematoxylin (blue) was used for nuclear staining (bright field image, 400× magnification). Kaplan–Meier estimates show a significantly shorter overall survival (*p* < 0.05) in patients with high expression of (**C**) ALKBH5 and (**F**) HNRNPC. Scale bar = 20 µm.

**Figure 3:**
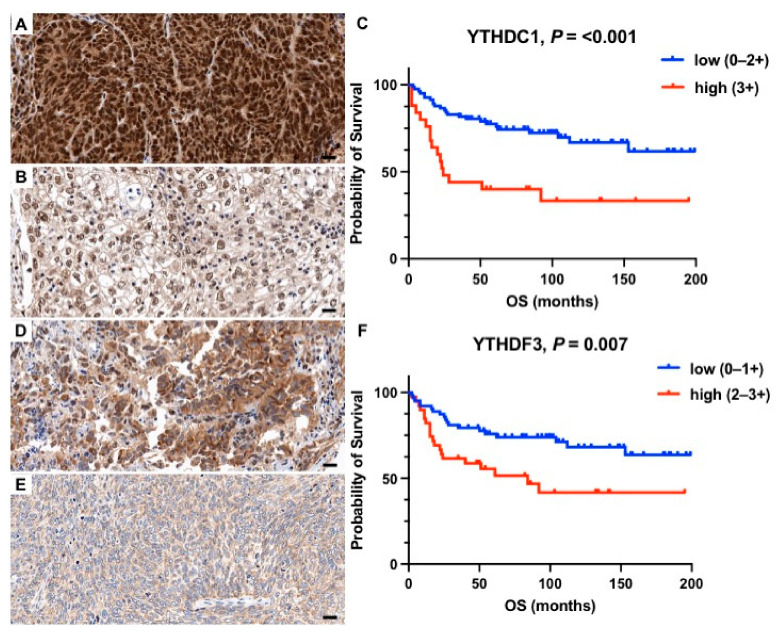
Representative histology sections show high (**A**,**D**) and low (**B**,**E**) expression levels of YTHDC1 and YTHDF3 visualized by immunohistochemistry; hematoxylin (blue) was used for nuclear staining (bright field image, 400× magnification). Kaplan–Meier estimates show a significantly shorter overall survival (*p* < 0.05) in patients with high expression of (**C**) YTHDC1 and (**F**) YTHDF3. Scale bar = 20 µm.

In Kaplan–Meier survival analysis, high expression levels of METTL14 (*p* = 0.016, Figure 1A–C), WTAP (*p* = 0.007, Figure 1D–F), KIAA1439 (*p* < 0.001, Figure 1G–I), ALKBH5 (*p* < 0.001, Figure 2A–C), HNRNPC (*p* = 0.012, Figure 2D–F), YTHDC1 (*p* < 0.001, Figure 3A–C), and YTHDF3 (*p* = 0.004, Figure 3D–F) correlated significantly with a shorter overall survival (OS). For the remaining proteins, there was a trend towards a shorter OS in patients with higher m6A protein expression levels, however, without reaching statistical significance (Table 2, Supplementary Figure S1). To correct for multiple hypothesis testing, the Benjamini and Hochberg method was applied with a significance threshold of *q* < 0.1. Prognostic significance remained after correction for multiple testing (*q* < 0.1) for the respective seven m6A proteins (Table 2). The prognostic value of METTL14, WTAP, KIAA1429, ALKBH5, HNRNPC, YTHDC1, and YTHDF3 was confirmed in univariate Cox regression analysis (Table 2). However, this prognostic value could not be observed in multivariate Cox regression analysis including established clinicopathological prognostic markers (age, grading, lymph node involvement, and FIGO stage).

Furthermore, correlation of m6A protein expression levels with respect to grading and lymph node involvement showed no statistically significant values.

**Figure 4:**
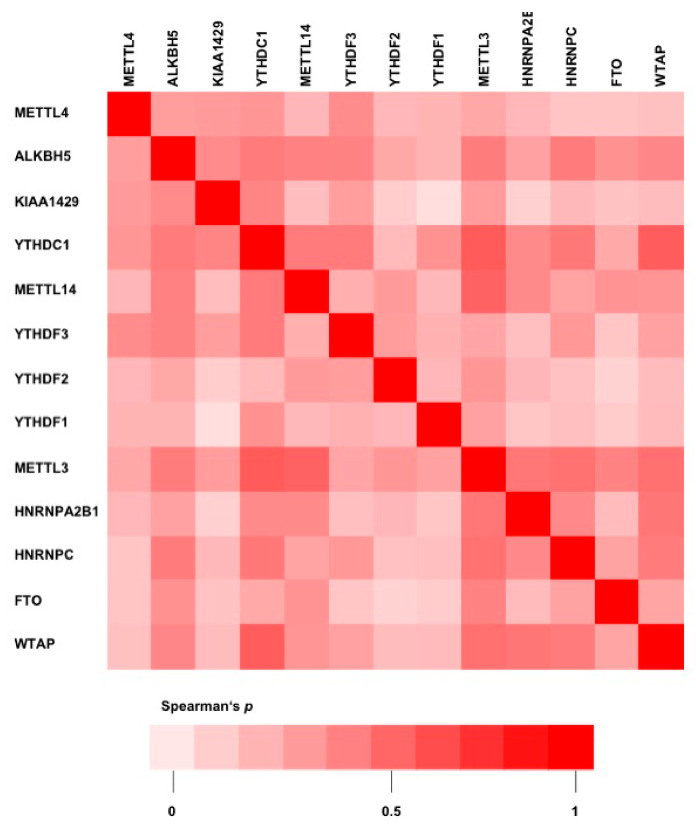
Correlation heatmap visualizing Spearman’s *p* correlation coefficients of m6A protein expression in the UHB CC cohort.

Of note, m6A protein expression levels showed high positive correlation coefficients towards each other, indicating a co-expression of proteins involved in m6A RNA modification in CC (Figure 4).

In the TCGA cohort, mRNA expression levels of *METTL14*, *WTAP*, *KIAA1429*, and *YTHDC1* were significantly associated with OS (*METTL14*: *p* = 0.012; *WTAP*: *p* = 0.041; *KIAA1429*: *p* = 0.016; *YTHDC1*: *p* = 0.026; Supplementary Table S2). In line with protein expression data obtained from the UHB CC cohort, enhanced mRNA expression levels of *METTL14*, *WTAP*, and *KIAA1429* were associated with a shorter OS. In contrast, enhanced *YTHDC1* mRNA expression was associated with a prolonged OS. However, after correction for multiple testing, the prognostic value of m6A mRNA expression did not reach statistical significance.

In summary, our results show that high protein expression levels of METTL14, WTAP, KIAA1429, ALKBH5, HNRNPC, YTHDC1, and YTHDF3 are associated with a shorter OS independent of their function (writer, reader, or eraser).

## 3. Discussion

In the present study, protein and mRNA expression levels of m6A writers, erasers, and readers were determined in two independent CC cohorts. mRNA and protein expression data were further analyzed with regard to clinical outcomes. On the protein level, we demonstrated that seven m6A proteins, namely METTL14, WTAP, KIAA1429, ALKBH5, HNRNPC, YTHDC1, and YTHDF3, were significantly associated with a poor OS in CC (UHB CC cohort; Table 2). In particular, higher expression levels of these respective proteins were linked to a shorter OS. Of note, this prognostic value was independent of lymph node involvement and tumor stage. These findings were substantiated by analyzing mRNA expression data obtained from an independent CC cohort (TCGA CC cohort). On the transcriptional level, we detected significant prognostic values for METTL14, WTAP, KIAA1429, and YTHDC1. In line with immunohistochemical data for METTL14, WTAP, and KIAA1429, higher mRNA levels were associated with a shortened OS. Contrasting results, however, were obtained for YTHDC1. Within the TCGA CC cohort, higher YTHDC1 mRNA expression levels were linked to prolonged OS, whereas in the UHB CC cohort, higher YTHDC1 protein expression levels were associated with a worse OS. Discordant expression data on the transcriptional and protein level are frequently reported in the literature and might be attributable to analytical issues and spatial tumor heterogeneity [13,14,15]. In the biological context, protein expression might be more relevant. However, caution is warranted when interpreting incongruent results obtained from two different cohorts. With regard to YTHDC1 and its prognostic value on OS in CC, further studies need to be conducted to clarify this issue.

There is broad scientific evidence that abnormal m6A modification plays an essential role in tumor proliferation, angiogenesis, and metastasis across various cancer types. In CC, m6A dysregulation was linked to chemo- and radiotherapy-resistance and a more progressive phenotype. Zhou et al. [16] reported enriched FTO expression in CC tumor tissue compared to normal adjacent tissue (NAT). Higher FTO expression levels were associated with enhanced resistance to chemo- and radiotherapy caused by decreased beta-catenin and increased ERCC1 expression levels. Another study indicated FTO as an important oncogenic driver in CC by regulating proliferation and migration of CC cells [17]. These findings are in line with data from our present study. In the UHB CC cohort, higher FTO protein expression showed a trend to a shortened OS in Kaplan–Meier survival analysis but without statistical significance (*p* = 0.061, Supplementary Figure S1). METTL3, in its function as a writer, was previously shown to be upregulated in CC cells. High METTL3 protein expression was correlated with a poor prognosis [18]. In our analyses, however, METTL3 expression levels had no distinct effect on OS (Table 2). Within the m6A writer subgroup, METTL14 is crucial for recognizing substrate RNAs and stabilizing the catalytic function of METTL3 [19,20]. A recent study on m6A in hepatocellular carcinoma (HCC) showed involvement of METTL4 in HCC tumor progression. In downstream analyses, this effect was attributed to m6A-dependent regulation of cysteine sulfidic acid decarboxylase (CSAD), glutamic-oxaloacetic transaminase 2 (GOT2), and suppressor of cytokine signaling 2 (SOCS2) [21]. In the UHB and TCGACC cohorts, METTL14 overexpression was identified to be associated with shortened OS. Analogous to METTL14, WTAP upregulation in HCC promoted liver cancer development [22]. Likewise, the same might be applicable to CC carcinogenesis. In both studied cohorts, the presence of WTAP overexpression was associated with worse OS. In our analyses, HNRNPC protein expression was identified among the m6A enzymes associated with poor OS. However, little is known regarding its role in carcinogenesis. Writers and erasers accomplish opposite functions. Hence, our finding of co-expression of these two enzyme groups appears to be counterintuitive (Figure 4). However, research has demonstrated a dual role for m6A in cancer biology comprising both cancer promotion and cancer suppression. Its specific role is dependent on the cell context and the downstream target RNA and its function (tumor promoter vs. tumor suppressor) [20,23,24]. In the literature, contradictory phenomena have been described for different tumor entities. While in breast cancer high FTO expression levels are associated with increased tumor cell proliferation, increased FTO levels in clear cell renal cell carcinoma resulted in tumor cell growth inhibition [25,26].

The YTH domain-containing proteins, including YTHDF1-3 and YTHDC1-2, participate in mRNA splicing, nuclear export, and translation. Due to post-transcriptional modifications, they modulate the expression of genes involved in cancer migration, invasion, proliferation, and immunity [27]. Especially unbalanced alternative splicing, which has been found in different kinds of cancer, can be caused by YTH domain dysregulation leading to tumor cell proliferation and invasion [28]. In our CC cohort, YTHDC1 and YTHDF3 overexpression was linked to shortened OS. Research has shown that nearly all YTH proteins, including YTHDF1-3 and YTHDC1-2, are upregulated in most types of cancer. In ovarian cancer, YTHDF1 facilitates tumorigenesis and metastasis by promoting the translation of EIF3C mRNA in an m6A-dependent manner [29]. In breast cancer, the overexpression of YTHDF1, YTHDF3, and KIAA1429 predicted a poor prognosis in terms of OS [30]. Furthermore, expression of YTHDC1, especially its alternative splicing components, was detected in a panel of prostate cell lines that was absent in benign cell lines, indicating that YTHDC1 might act as an oncogene in prostate cancer [31].

As m6A RNA modification is implicated in carcinogenesis, it might display a potential target for anticancer therapy. In dendritic cells, loss of YTHDF1 enhanced the cross-presentation of tumor antigens and the cross-priming of CD8(+) T cells in vivo. Furthermore, the therapeutic efficacy of the PD-L1 checkpoint was enhanced in YTHDF1 (−/−) mice, indicating that YTHDF1 might be a potential therapeutic target in anticancer immunotherapy [32]. In colorectal cancer and melanoma, loss of METTL3 and METTL14 enhanced the sensitivity to anti-PD-1 treatment [33]. ALKBH5 regulates the content of lactic acid and accumulation of tumor immune cells in the tumor microenvironment, so that ALKBH5 might serve as a potential therapeutic target to enhance the effect of immunotherapy in melanoma, colorectal, and potentially other cancer types [34]. The influence of m6A proteins on targeted cancer therapy, especially checkpoint inhibitors, might also have an impact in CC patients, where PD-L1 inhibitors are used for the therapy in the recurrent or metastatic setting [35].

Overall, these findings point towards the potential impact of m6A RNA modification for CC and cancer in general. Limitations of our study are the retrospective design. Protein expression analysis is based on tissue microarray, where tumor heterogeneity might be a potential bias. However, within our study, clinically relevant signals were detected. The dysregulation of m6A proteins might be used as biomarkers and indicators for poor prognosis but also as potential targets for novel therapeutic drugs. There is still a need to conduct further studies to investigate their biological functions and precise corresponding molecular mechanisms in detail.

## 4. Materials and Methods

### 4.1. Patients and Specimens

UHB CC cohort: The retrospective study population comprised 118 patients with CC diagnosed at the University Hospital between 2002 and 2016. The collection of tissue was performed within the framework of the Biobank initiative of the University Hospital Bonn. Tissue was obtained from biopsies or surgical specimens. All patients provided written informed consent prior to collection of biomaterials. The study was approved by the Ethics Committee of the University of Bonn (vote: 208/21).

Clinicopathological parameters are summarized in Table 1. Baseline characteristics were obtained from a clinical database. Histopathological diagnosis was deduced based on World Health Organization (WHO) criteria. The International Federation of Gynecology and Obstetrics (FIGO) classification was used to determine the tumor stage.

TCGA CC cohort: mRNA expression data from 307 CC patients were obtained from The Cancer Genome Atlas Research Network [36]. Patients had signed informed consent prior to registration in accordance with the declaration of Helsinki principles. Clinicopathological characteristics of this cohort have been published elsewhere [37].

### 4.2. Tissue Microarray (TMA) Construction (UHB CC Cohort)

Formalin-fixed paraffin-embedded CC tissue (FFPE) specimens were used to generate TMAs. Staining with hematoxylin and eosin (HE) was performed to identify representative tumor areas. For each case, two 1 mm core biopsies (0.785 mm^2^) were taken from different cancer areas and arranged in TMA blocks.

### 4.3. Immunohistochemistry

Immunostaining of the different writers, erasers, and readers was performed on TMAs. An automated staining system (BenchMark ULTRA; Ventana Medical Systems, Oro Valley, AZ, USA) was applied for deparaffinization, pretreatment with cell conditioning buffer (CC1 buffer, pH8), and primary antibody incubation. Incubation with the primary antibody was performed at 4 °C overnight. For signal detection, the UltraView DAB IHC Detection Kit (Ventana) was used. A detailed overview of the antibodies and dilutions is presented in Supplementary Table S1.

For immunohistochemical analyses, an Olympus BX51 microscope (Olympus, Tokio, Japan) and the Panoramic Viewer 3DHistech (3DHISTECH Kft., Budapest, Hungary) were used. Staining intensities were evaluated by two different investigators on technical duplicates. Briefly, a four-tier scoring system was applied to categorize staining intensities (0: no staining, 1: low staining, 2: moderate staining, 3: high staining). The obtained staining intensities were divided into two groups (low and high) with the median protein expression as a cut-off. For ALKBH5 and YTHDC1, immunohistochemical staining of multiple subcellular compartments was observed. Here, the predominant subcellular localization (nuclear) was considered for statistical analysis. Classification of the groups, low vs. high depending on the staining intensity, is provided for each antibody in Table 2.

### 4.4. Statistical Analysis

Statistical analysis (Kaplan–Meier survival analysis, log-rank tests, Cox regression analysis, and non-parametric Spearman’s *p* correlation coefficients) were performed with the Statistical Package for the Social Sciences (SPSS^®^) version 28 (SPSS INC., IBM Corp., Armonk, NY, USA) and the GraphPad Prism software (GraphPad Software, San Diego, CA, USA). Statistical significance was approved at a two-sided *p* < 0.05. To correct for multiple testing, the Benjamini and Hochberg method was applied. *P*-values were converted to false discovery rate (FDR) *Q*-values with a significance threshold of *q* < 0.1.

## 5. Conclusions

In CC, enhanced expression levels of m6A proteins are associated with unfavorable clinical outcomes. This effect is independent of established clinicopathological prognostic parameters. Hence, our study highlights the potential of m6A as a promising prognostic biomarker in CC. The crucial role of m6A in CC pathogenesis holds the potential for the development of new anticancer therapeutics.

## Figures and Tables

**Table 1:** Clinicopathological characteristics of the UHB CC cohort (*N* = 118). SD = standard deviation. IHC = immunohistochemistry.

Cervical Cancer Cohort	
**Age (years)**	
Mean (±SD)	51.3 ± 13.9
Min–max	21–88
**Histology**	
Squamous cell carcinoma	98 (83.1%)
Adenocarcinoma	20 (16.9%)
**Follow-up (months)**	
Mean (±SD)	77.58 ± 58.3
Min–max	0–199
**FIGO classification**	
IA	5 (4.2%)
IB	56 (47.5%)
IIA	11 (9.3%)
IIB	18 (15.3%)
III	13 (11.0%)
IVA	15 (12.7%)
**Lymph node involvement**	
Yes	26 (22.0%)
No	54 (45.8%)
Unkown	38 (32.2%)
**Grading**	
G1	2 (1.7%)
G2	76 (64.4%)
G3	39 (33.1%)
Unknown	1 (0.8%)
**HPV-Status (p16 IHC positive)**	
Positive	107 (90.7%)
Negative	5 (4.2%)
Unknown	6 (5.1%)

**Table 2:** Summary of analyzed proteins and their correlation with OS in the UHB CC cohort. *p*-values for the group comparison (low vs. high expression) are based on log-rank tests, significance threshold *p* < 0.5, and estimated hazard ratios (HR) with 95% confidence intervals are based on univariate Cox regression analyses, significance threshold *p* < 0.5. *Q*-values are based on multiple hypotheses testing using the method of Benjamini and Hochberg with a significance threshold of *q* < 0.1. Significant values are highlighted in bold.

Proteins	Localization	Staining Intensity	N (Low/High)	*p*-Value (log)	*q*-Value	Hazard Ratio	95% CI	*p*-Value (cox)
**Writer**								
METTL3	Nuclear	0–1+/2–3+	15/92	0.128	0.166	2.416	0.745–7.830	0.142
METTL4	Cytoplasmatic	0–1+/2–3+	49/61	0.448	0.448	1.264	0.689–2.319	0.450
METTL14	Nuclear	0–1+/2–3+	31/82	**0.016**	**0.030**	2.592	1.154–5.825	**0.021**
WTAP	Nuclear	0–2+/3+	90/20	**0.007**	**0.018**	2.387	1.239–4.598	**0.009**
KIAA1429	Nuclear	0–1+/2–3+	96/13	**<0.001**	**0.013**	5.838	2.886–11.812	**<0.001**
**Eraser**								
FTO	Nuclear	0–1+/2–3+	36/75	0.061	0.100	2.060	0.951–4.462	0.067
ALKBH5	Cytoplasmatic/ nuclear	0–1+/2–3+	60/51	**<0.001**	**0.004**	3.603	1.837–7.068	**<0.001**
**Reader**								
HNRNPA2B1	Nuclear	0–2+/3+	75/37	0.108	0.156	1.628	0.892–2.972	0.112
HNRNPC	Nuclear	0–1+/2–3+	41/66	**0.012**	**0.026**	2.506	1.196–5.254	**0.015**
YTHDC1	Membraneous/cytoplasmatic/ nuclear	0–2+/3+	82/26	**<0.001**	**0.007**	3.284	1.758–6.134	**<0.001**
YTHDF1	Cytoplasmatic	0–2+/3+	76/33	0.206	0.243	1.522	0.789–2.936	0.210
YTHDF2	Cytoplasmatic	0–2+/3+	89/21	0.260	0.281	1.499	0.737–3.051	0.264
YTHDF3	Cytoplasmatic	0–1+/2–3+	63/40	**0.004**	**0.013**	2.422	**1.289–4.550**	**0.006**

## Data Availability

Raw data are available on request from the authors.

## Supplementary Materials

The following supporting information can be downloaded at: https://www.mdpi.com/article/10.3390/ijms23137165/s1.
