# Supplementary material for: Comprehensive Analysis of N6-Methyladenosine (m6A) Writers, Erasers, and Readers in Cervical Cancer
Source: Int J Mol Sci. 2022 Jun 28;23(13):7165. doi: 10.3390/ijms23137165 (PMC9266832; doi:10.3390/ijms23137165)
Supplement: Supplementary file 1 [file ijms-23-07165-s001.zip › ijms-1759226-supplementary.pdf]

Protein	Dilution	Company
**Writer**		
METTL3	1:1000	Biorbyt #orb374082
METTL4	1:200	Novus #NBP1-82188
METTL14	1:100	Atlas Antibodies #HPA038002
WTAP	1:100	Atlas Antibodies #HPA010550
KIAA1429	1:25	Atlas Antibodies #HPA031530
**Eraser**		
FTO	1:50	Atlas Antibodies #HPA041086
ALKBH5	1:200	Novus #NBP1-82188
**Reader**		
HNRNPA2B1	1:100	Atlas Antibodies #HPA001666
HNRNPC	1:25	Atlas Antibodies #HPA051075
YTHDC1	1:25	Atlas Antibodies #HPA036462
YTHDF1	1:10	Biorbyt #orb179018
YTHDF2	1:200	Biorbyt #orb39199
YTHDF3	1:200	Biorbyt #orb374095

Table S1. Overview of antibodies and dilutions.

mRNA	*p*- value (log rank)	*q*-value	Hazard ratio	95% CI	*p*- value (cox)
**Writer**					
METTL3	0.497	0,718	1.172	0.740-1.855	0.498
METTL4	0.754	0,817	0.929	0.587-1.472	0.754
METTL14	**0.012**	0,156	1.814	1.129-2.915	**0.014**
WTAP	**0.041**	0,133	1.625	1.016-2.600	**0.043**
KIAA1429	**0.016**	0,104	1.760	1.106-2.800	**0.017**
**Eraser**					
FTO	0.801	0,801	1.061	0.670-1.681	0.801
ALKBH5	0.486	0,79	0.849	0.536-1.346	0.487
**Reader**					
HNRNPA2B1	0.091	0,197	1.487	0.936-2.361	0.093
HNRNPC	0.674	0,476	0.906	0.571-1.436	0.674
YTHDC1	**0.026**	0,113	0.592	0.371-0.944	**0.028**
YTHDF1	0.272	0,505	0.773	0.487-1.226	0.274
YTHDF2	0.674	0,797	1.104	0.697-1.748	0.674
YTHDF3	0.051	0,133	1.578	0.995-2.503	0.053

Table S2. Summary of analyzed mRNAs and their correlation with OS. *P*-values for the group comparison (low vs., high expression by median split) are based on log-rank tests, significance threshold $p<0.5$, estimated hazard ratios (HR) with 95% confidence intervals are based on univariate Cox regression analyses, significance threshold $p<0.5$. *Q*-values are based on multiple hypotheses testing using the method of Benjamini and Hochberg with a significance threshold of $q<0.1$.

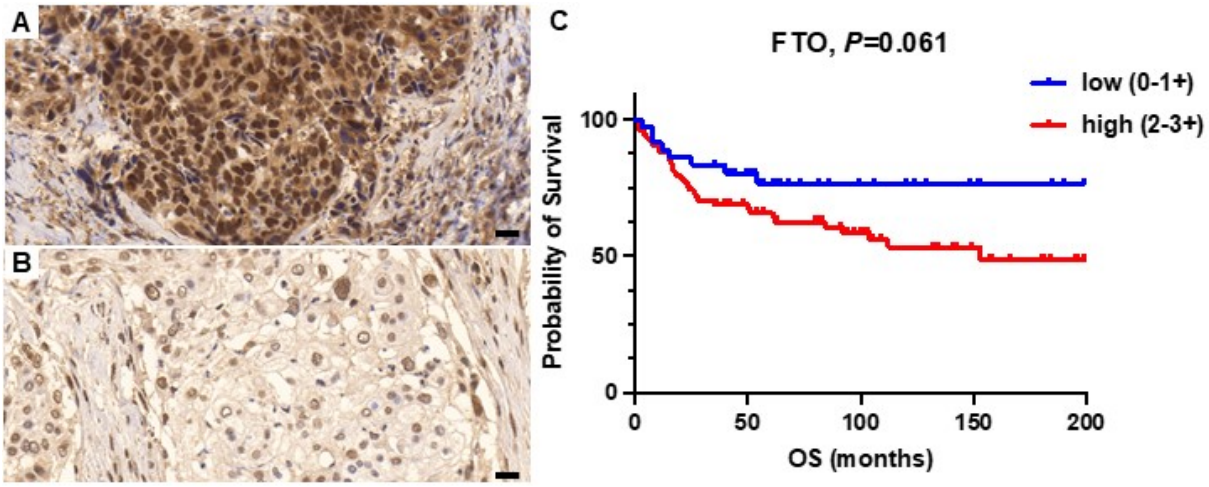

Figure S1. Representative histology sections show high (A) and low (B) expression levels of FTO visualized by immunohistochemistry; hematoxylin (blue) was used for nuclear staining (bright field image, 400x magnification). Kaplan-Meier estimates show a trend towards a shorter overall survival in patients with high expression of FTO. Scale bar = 20 µm.

METTL14, high; 400x

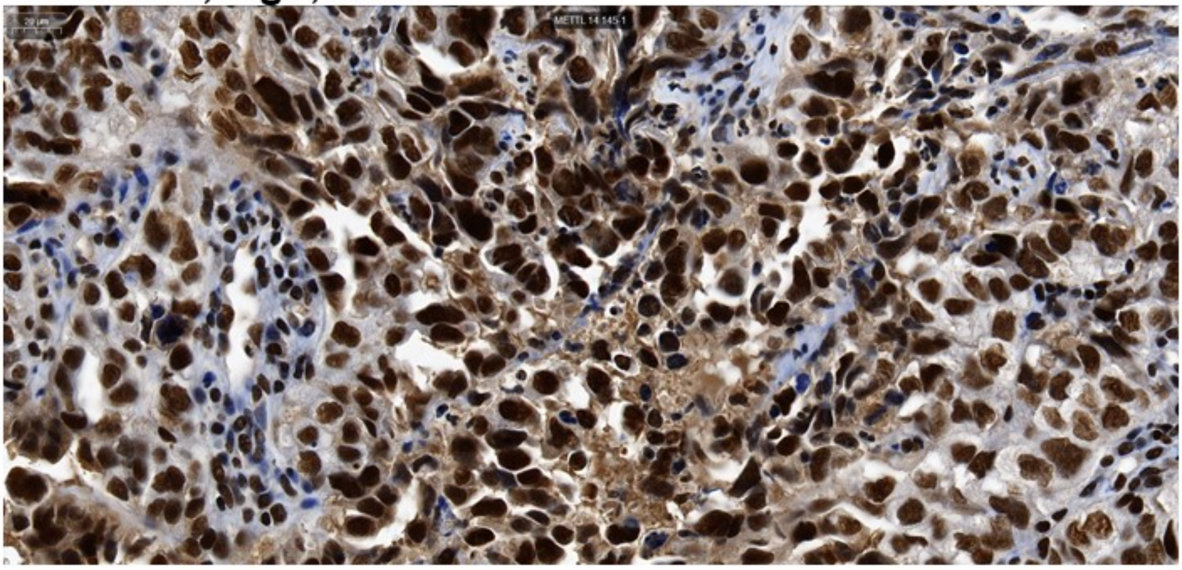

METTL14, low; 400x

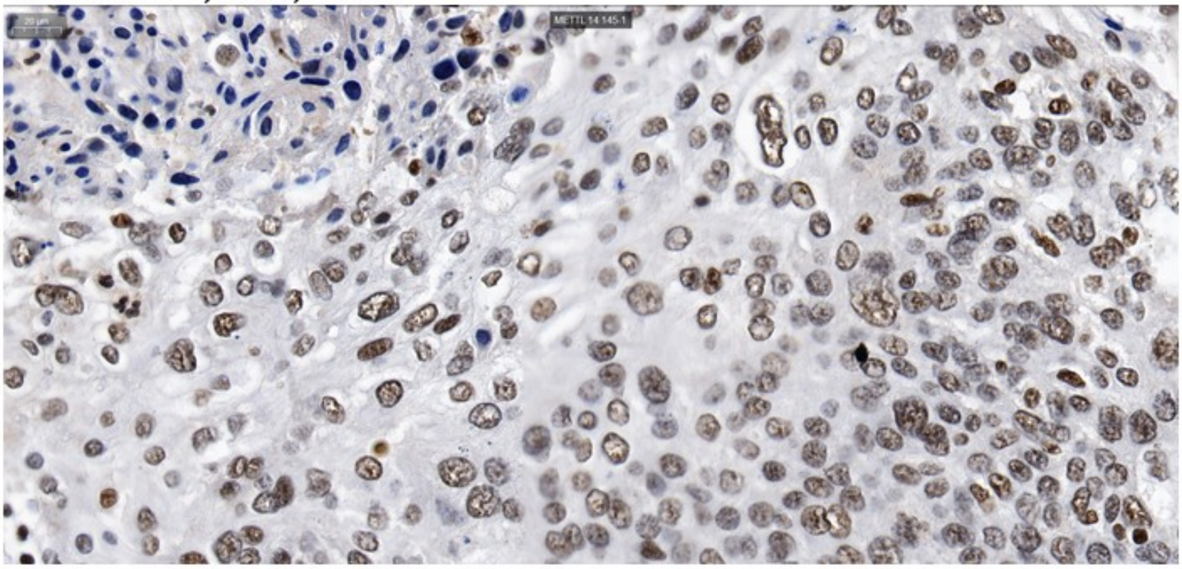

Figure S2. Representative histology sections show high (upper panel) and low (lower panel) expression levels of METTL14 visualized by immunohistochemistry; hematoxylin (blue) was used for nuclear staining (bright field image, 400x magnification). Scale bar = 20 µm.

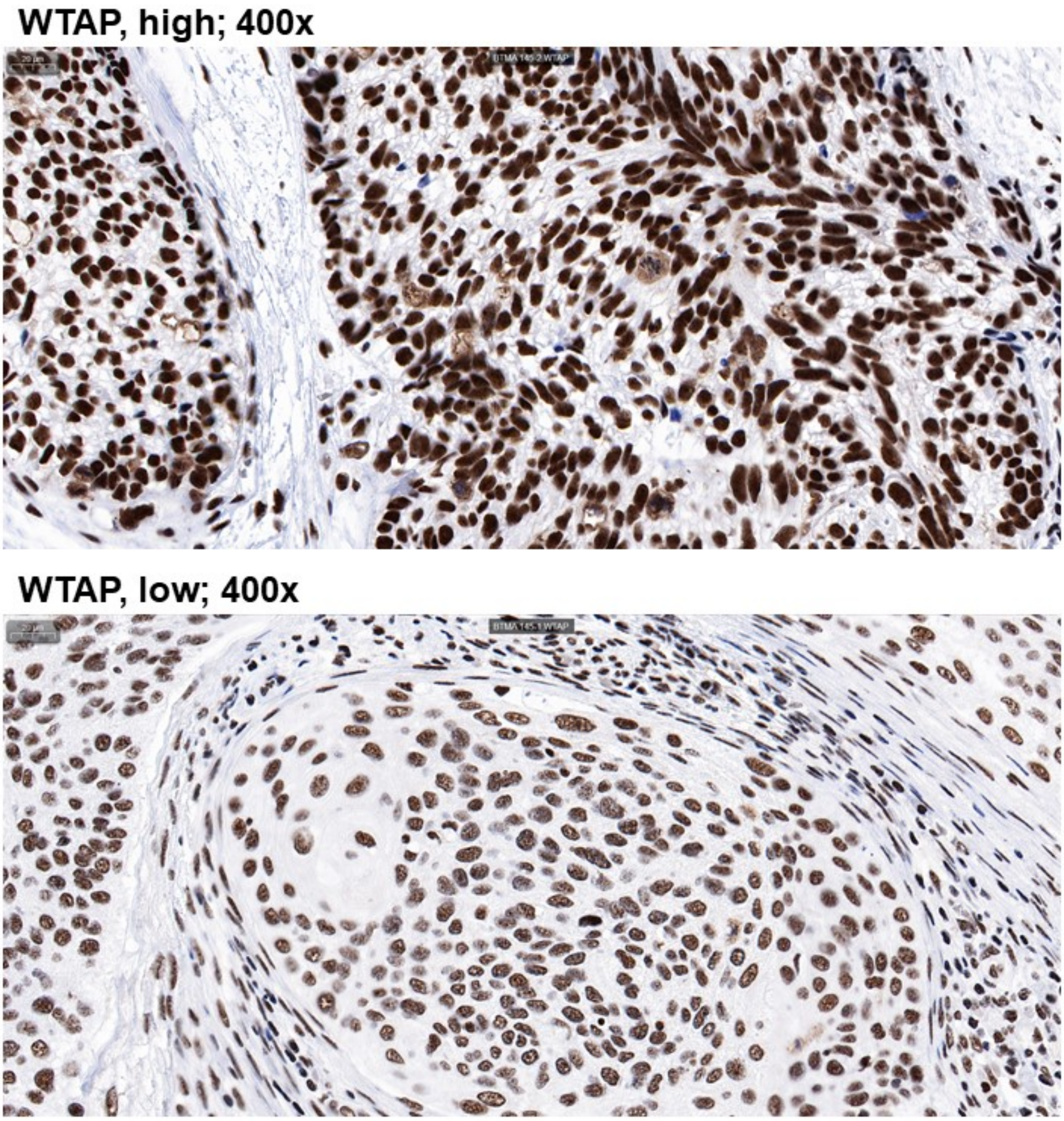

Figure S3. Representative histology sections show high (upper panel) and low (lower panel) expression levels of WTAP visualized by immunohistochemistry; hematoxylin (blue) was used for nuclear staining (bright field image, 400x magnification). Scale bar = 20 µm.

KIAA1429, high; 400x

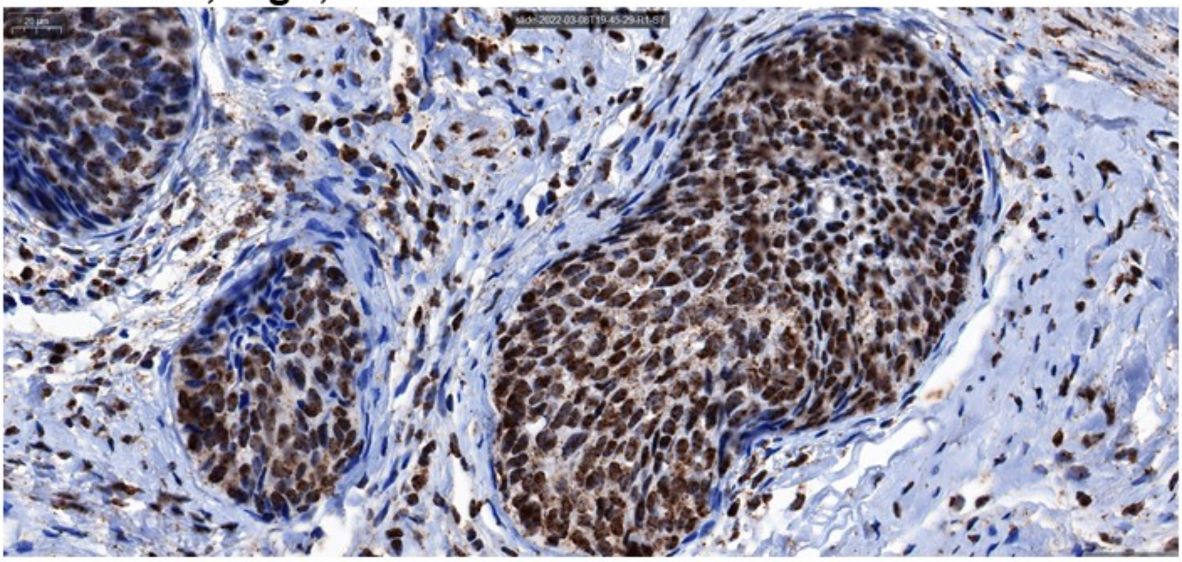

KIAA1429, low; 400x

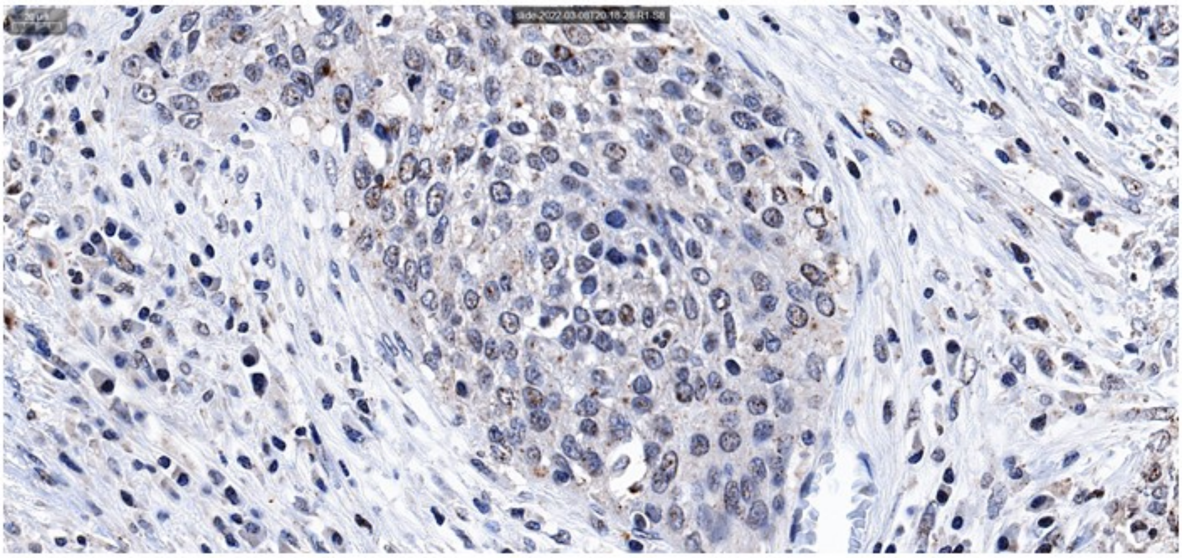

Figure S4. Representative histology sections show high (upper panel) and low (lower panel) expression levels of KIAA1429 visualized by immunohistochemistry; hematoxylin (blue) was used for nuclear staining (bright field image, 400x magnification). Scale bar = 20 µm.

ALKBH5, high; 400x

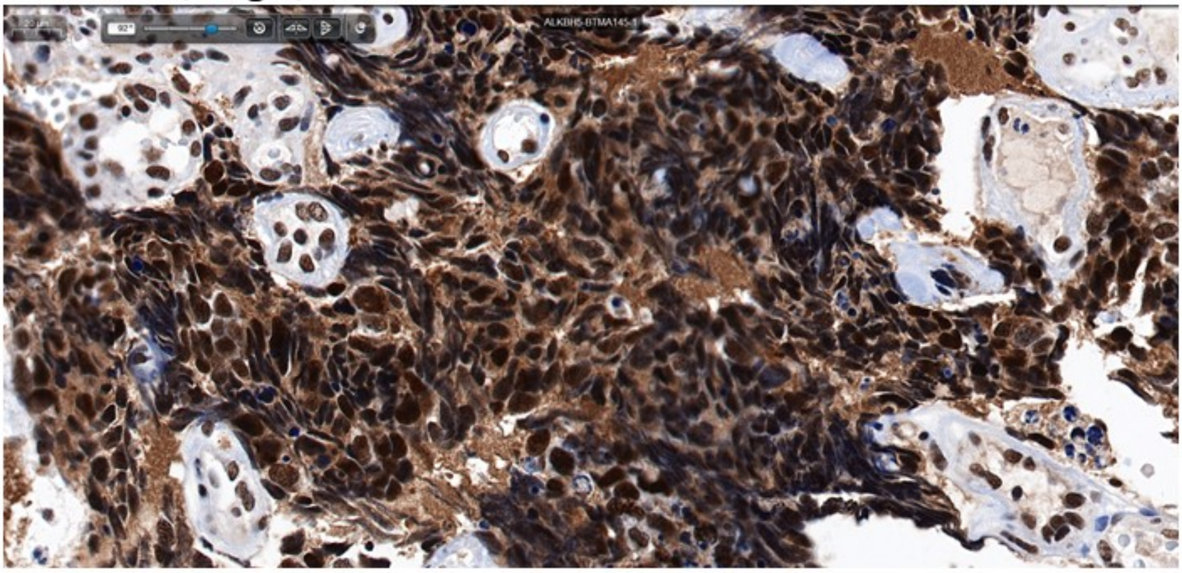

ALKBH5, low; 400x

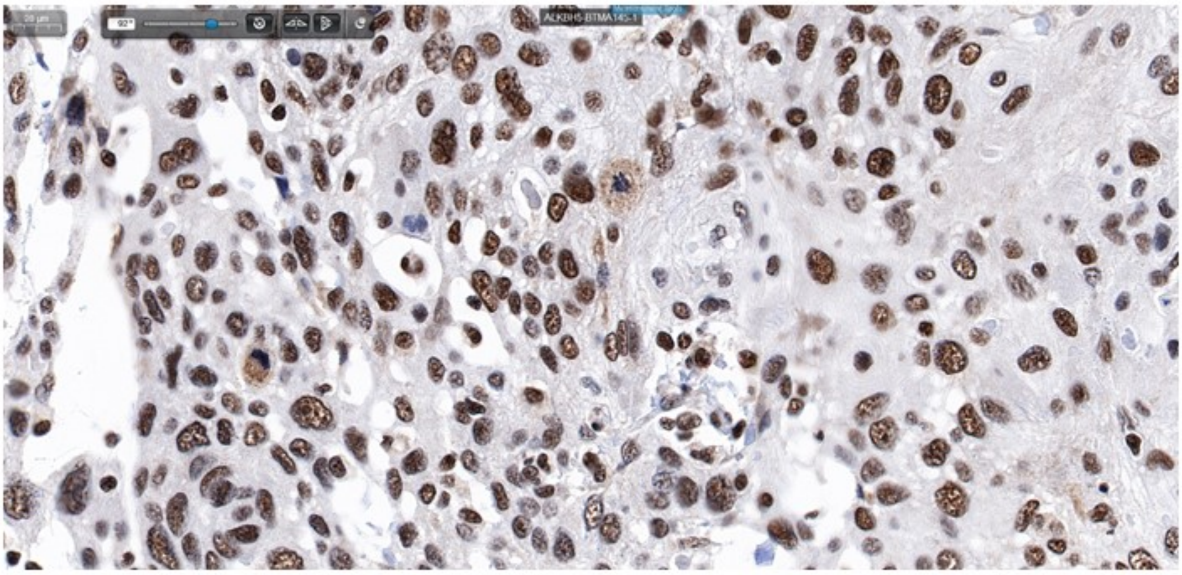

Figure S5. Representative histology sections show high (upper panel) and low (lower panel) expression levels of ALKBH5 visualized by immunohistochemistry; hematoxylin (blue) was used for nuclear staining (bright field image, 400x magnification). Scale bar = 20 µm.

HNRNPC, high; 400x

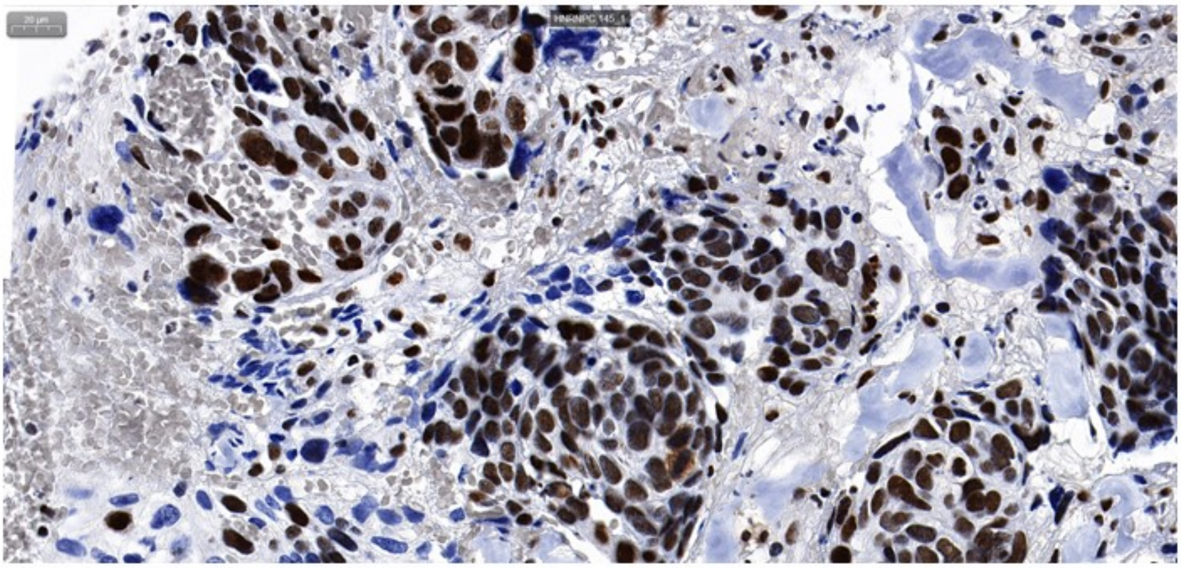

HNRNPC, low; 400x

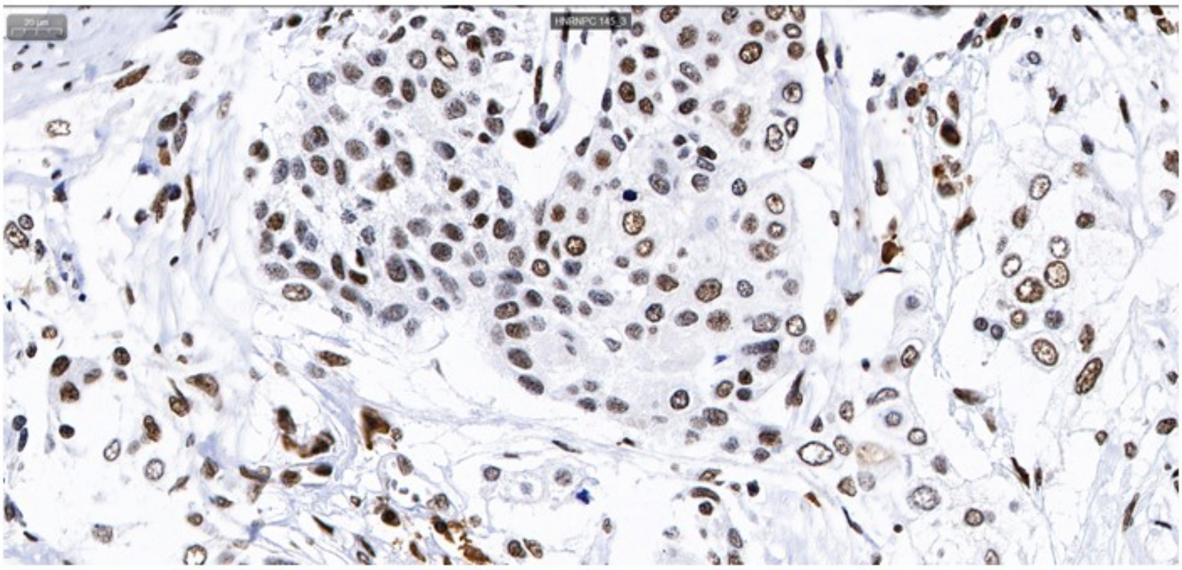

Figure S6. Representative histology sections show high (upper panel) and low (lower panel) expression levels of HNRNPC visualized by immunohistochemistry; hematoxylin (blue) was used for nuclear staining (bright field image, 400x magnification). Scale bar = 20 μm.

YTHDC1, high; 400x

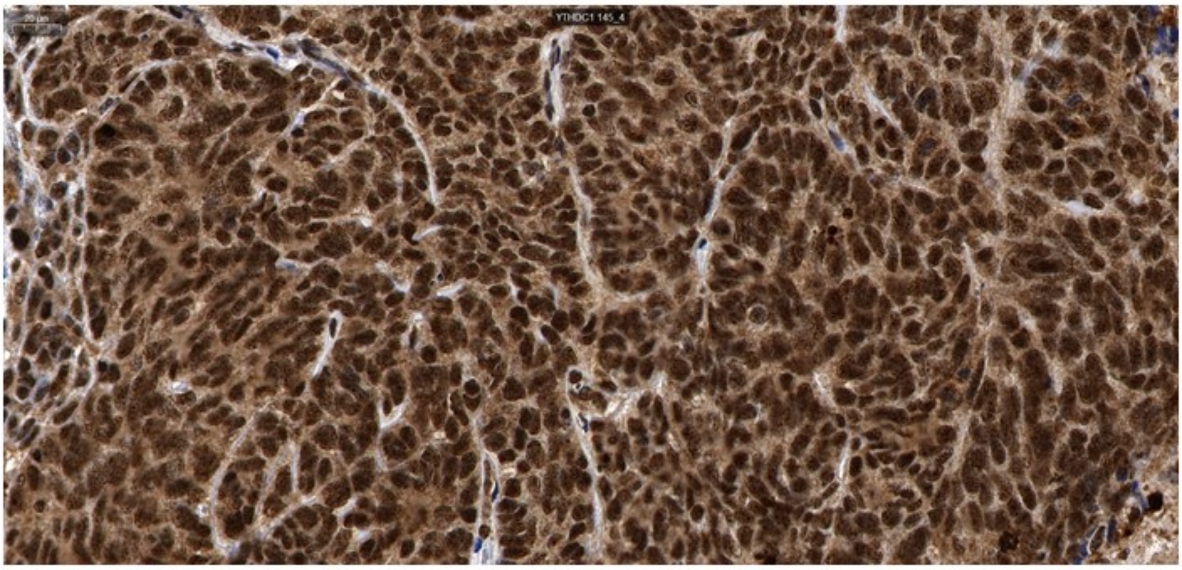

YTHDC1, low; 400x

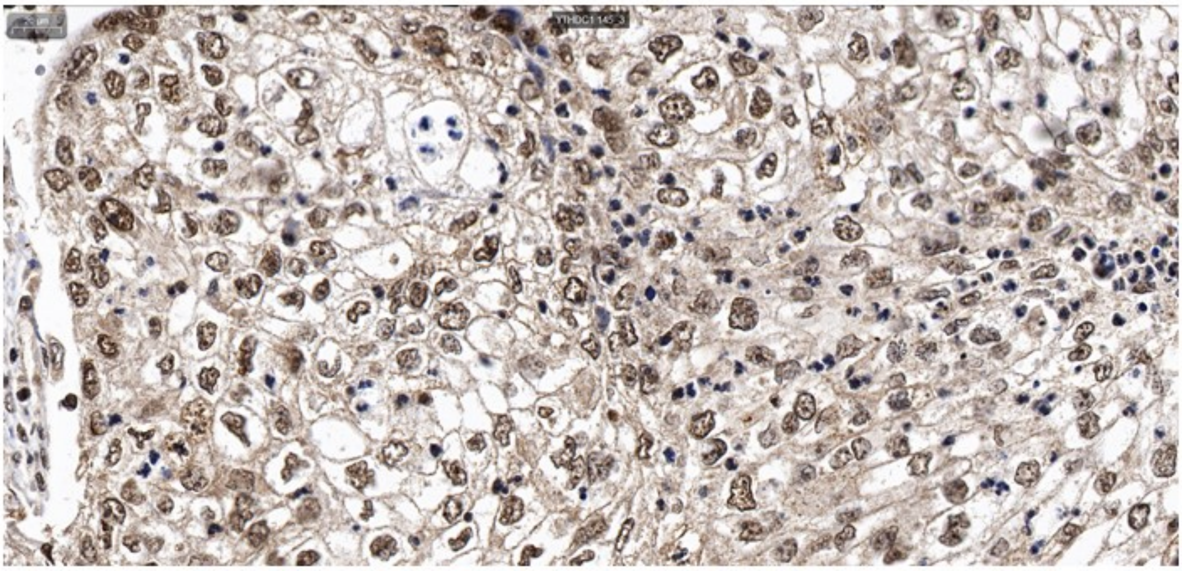

Figure S7. Representative histology sections show high (upper panel) and low (lower panel) expression levels of YTHDC1 visualized by immunohistochemistry; hematoxylin (blue) was used for nuclear staining (bright field image, 400x magnification). Scale bar = 20 µm.

YTHDF3, high; 400x

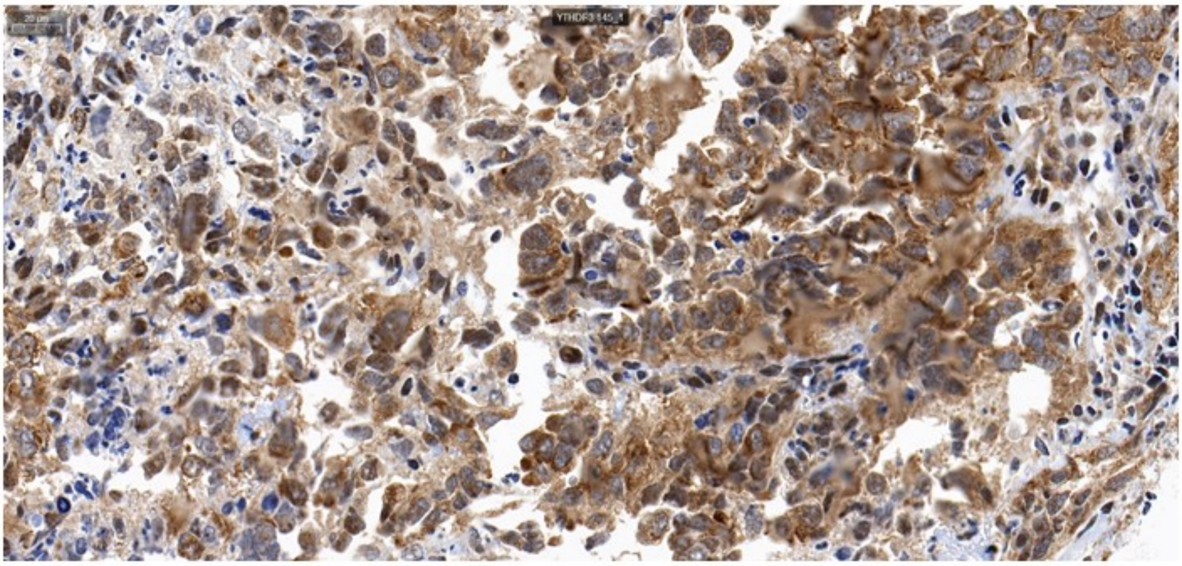

YTHDF3, low; 400x

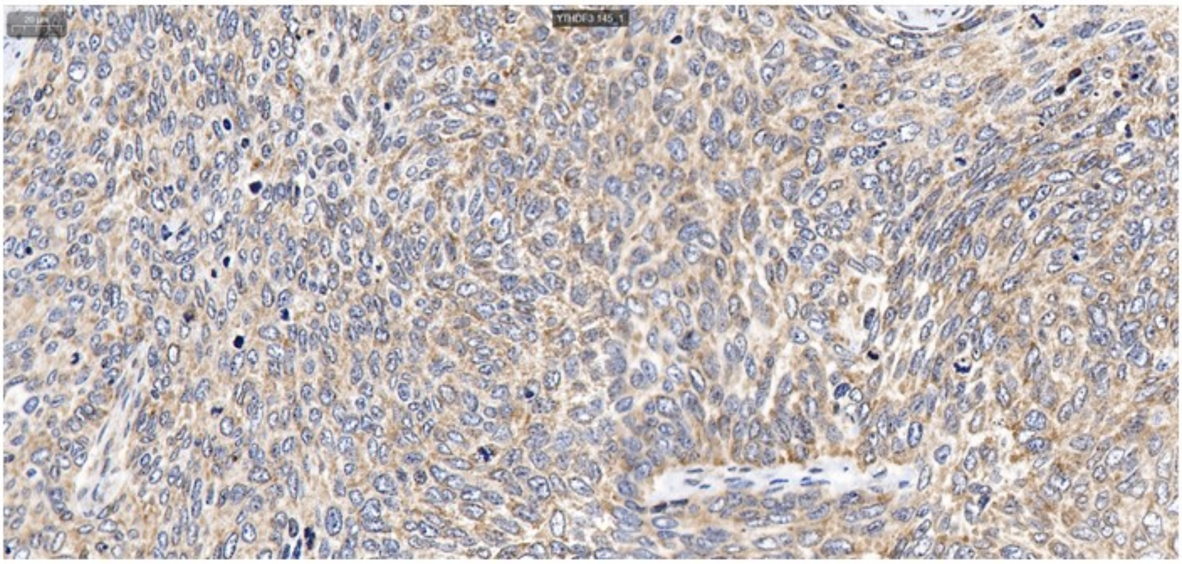

Figure S8. Representative histology sections show high (upper panel) and low (lower panel) expression levels of YTHDF3 visualized by immunohistochemistry; hematoxylin (blue) was used for nuclear staining (bright field image, 400x magnification). Scale bar = 20 µm.
